# Influence of Chest Compressions on Circulation during the Peri-Cardiac Arrest Period in Porcine Models

**DOI:** 10.1371/journal.pone.0155212

**Published:** 2016-05-11

**Authors:** Jun Xu, Chen Li, Yan Li, Joseph Walline, Liangliang Zheng, Yangyang Fu, Dongqi Yao, Huadong Zhu, Xiaohe Liu, Yanfen Chai, Zhong Wang, Xuezhong Yu

**Affiliations:** 1 Emergency Department, Peking Union Medical College Hospital, Chinese Academy of Medical Sciences, Beijing, China; 2 Emergency Department, Tianjin Medical University General Hospital, Tianjin, China; 3 Division of Emergency Medicine, Department of Surgery, Saint Louis University Hospital, Saint Louis, Missouri, United States of America; 4 Emergency Department, Beijing Tsinghua Changgung Hospital, Beijing, China; Azienda Ospedaliero-Universitaria Careggi, ITALY

## Abstract

**Objective:**

Starting chest compressions immediately after a defibrillation shock might be harmful, if the victim already had a return of spontaneous circulation (ROSC) and yet was still being subjected to external compressions at the same time. The objective of this study was to study the influence of chest compressions on circulation during the peri-cardiac arrest period.

**Design:**

Prospective, randomized controlled study.

**Setting:**

Animal experimental center in Peking Union Medical Collage Hospital, Beijing, China.

**Subjects:**

Healthy 3-month-old male domestic pigs.

**Interventions:**

44 pigs (28±2 kg) were randomly assigned to three groups: Group I (non-arrested with compressions) (n = 12); Group II (arrested with compressions only) (n = 12); Group III (ROSC after compressions and defibrillation) (n = 20). In Groups I and II, compressions were performed to a depth of 5cm (Ia and IIa, n = 6) or a depth of 3cm (Ib and IIb, n = 6) respectively, while in Group III, the animals which had just achieved ROSC (n = 18) were compressed to a depth of 5cm (IIIa, n = 6), a depth of 3cm (IIIb, n = 6), or had no compressions (IIIc, n = 6). Hemodynamic parameters were collected and analyzed.

**Measurements and Findings:**

Hemodynamics were statistically different between Groups Ia and Ib when different depths of compressions were performed (p < 0.05). In Group II, compressions were beneficial and hemodynamics correlated with the depth of compressions (p < 0.05). In Group III, compressions that continued after ROSC produced a reduction in arterial pressure (p < 0.05).

**Conclusions:**

Chest compressions might be detrimental to hemodynamics in the early post-ROSC stage. The deeper the compressions were, the better the effect on hemodynamics during cardiac arrest, but the worse the effect on hemodynamics after ROSC.

## Introduction

Sudden cardiac arrest is still a leading cause of death around the world[1]. Chest compressions (CCs) are the foundation of cardiopulmonary resuscitation (CPR). The latest European Resuscitation Council (ERC) /American Heart Association(AHA) CPR guidelines emphasized the importance of high quality CCs, including an adequate rate (100-120/minute), adequate depth (5-6cm), adequate chest recoil after each compression, and minimizing interruptions in compressions [2, 3]. All the measures above can help maintain temporary artificial circulation to the heart, brain and other key organs, hopefully achieving return of spontaneous circulation (ROSC) and patient survival. Multiple studies show that high quality CCs are critical during CPR [4, 5].

However, CCs after ROSC may be harmful. The atria and ventricles compress sequentially in the normal physiologic cardiac cycle, while CCs which compress the four chambers simultaneously disturb this pattern. Additionally, the rate of CCs seldom matches the inherent heart rate during spontaneous circulation. Some studies have shown that the mechanical force generated by CCs after ROSC in humans could lead to ventricular re-fibrillation [6, 7]. Therefore, we wonder if performing CCs after ROSC may be causing more harm than good.

This randomized laboratory investigation studied the influence of CCs on hemodynamic parameters during the peri-arrest period of cardiac arrest (CA) induced by ventricular fibrillation (VF) in porcine models.

## Materials and Methods

### Animal Preparation

This experimental protocol was approved by the Animal Care and Use Committee at Peking Union Medical College Hospital (2013S-512).

44 healthy 3-month-old male domestic swine [(28±2)kg] were fasted overnight, and then anesthetized by 3% Pentobarbital Sodium (Merck, 719F034, Germany) 1ml/kg IM followed by inhalational 4% isoflurane (ABBOTT, H20059911, USA) via a snout mask with 100% oxygen using an anesthesia apparatus (Veterinary Anesthesia Ventilator, Midmark Corporation, USA). Anesthesia was maintained with intravenous propofol (2 mg/(kg∙h)) (Corden Pharma S.P.A., H20100645, Italy) after endotracheal intubation and mechanical ventilation initiation. The animals were anesthetized throughout the whole duration of the study until euthanasia. Volume control mode without PEEP was given (tidal volume = 8~10ml/kg, Rate = 10/min) by mechanical ventilator (Esprit Ventilato, V1000, Germany). The tidal volume was adjusted to maintain partial pressure of end-tidal carbon dioxide (P_ET_CO_2_) at 35~45 mmHg.

The pigs were placed in a specially designed adjustable U-shaped fixing frame in supine position to avoid tube dislocation or movement during CCs. The right femoral artery in each swine was cannulated with 4-Fr thermo dilution PiCCO catheter (Pulsion Medical Systems AG, Munich, Germany) and connected to the monitor (T8, Mindray, Shenzhen, China) to continuously monitor the hemodynamic parameters. A central venous catheter (18Ga, 20 cm, Arrow, USA) was inserted through the right internal jugular vein into the right atria of each animal for right atrial pressure (RAP) measurements. Another central venous catheter was inserted into the left internal jugular vein in each animals to allow passage of an endocardial electrode catheter to induce VF by 24V /50 HZ alternating current lasting 1-second.

### Experimental Protocol

**Fig 1:**
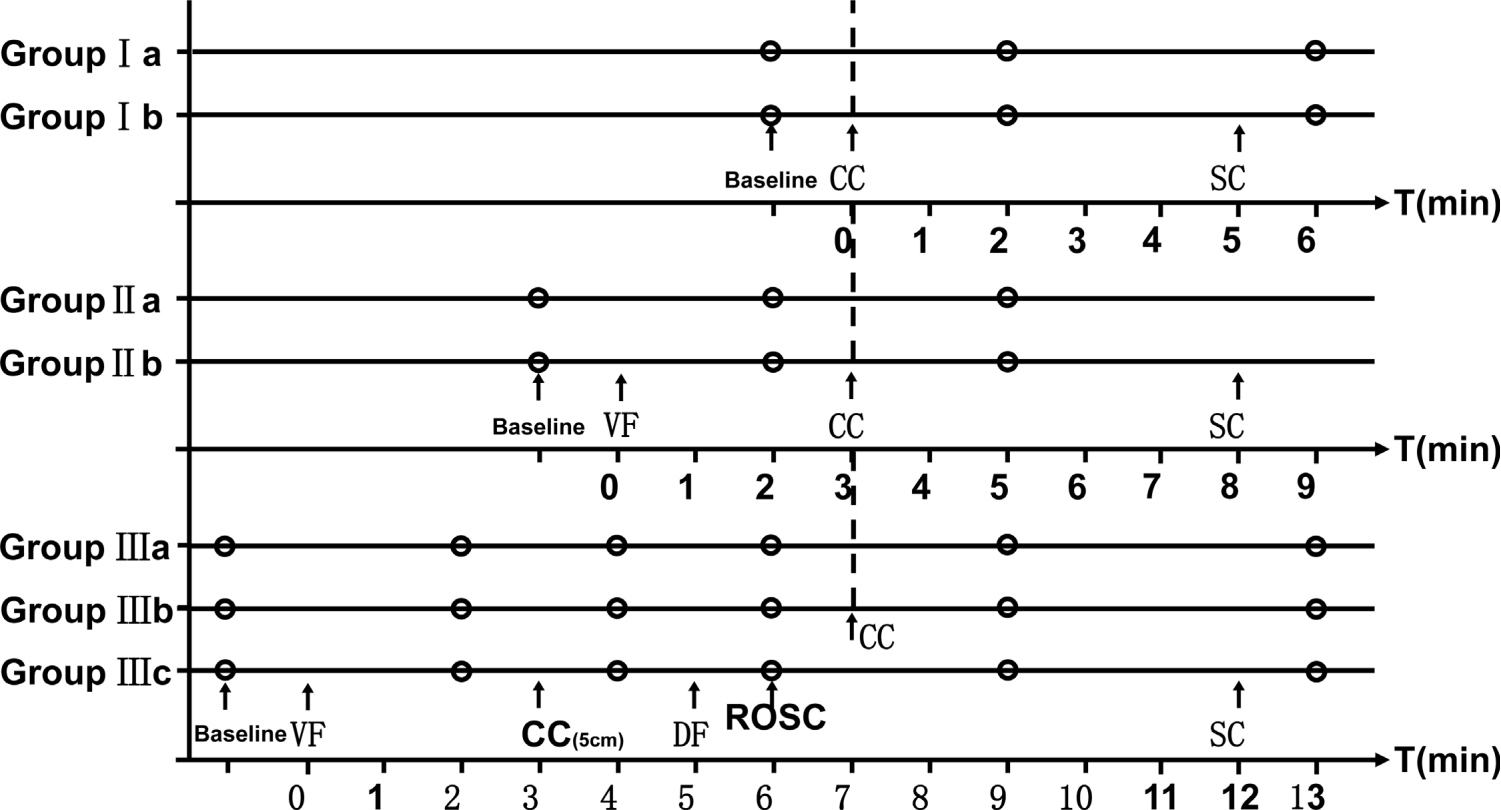
Experimental Protocol. Group Ia = non-arrested with chest compressions (to a depth of 5cm); Group Ib = non-arrested with chest compressions (to a depth of 3cm); Group IIa = arrested with chest compressions (to a depth of 5cm) only; Group IIb = arrested with chest compressions (to a depth of 3cm) only; Group IIIa = compressions to a depth of 5cm continued after ROSC; Group IIIb = compressions to a depth of 3cm continued after ROSC; Group IIIc = chest compressions stopped after ROSC; ROSC = return of spontaneous circulation; VF = ventricular fibrillation; CC = chest compressions; DF = defibrillation; SC = stop compressions.

44 male pigs (28±2 kg) were randomly assigned to three groups: Group I (non-arrested with CCs) (n = 12), Group II (arrested with CCs only) (n = 12), and Group III (ROSC after CCs and defibrillation) (n = 18) (Fig 1).

In Group I, CCs were performed on the animals to a depth of 5cm (Ia, n = 6) or a depth of 3cm (Ib, n = 6) for a 5-minute duration 30-minutes after animal preparation. The rate and depth of CCs were controlled by a CPR machine (WISH-SL-FS-A, Wuhan, China). Hemodynamic parameters were collected at initiation and at the beginning of the second and sixth minutes.

In Group II, again 30-minutes after animal preparation, VF was induced. After 3 minutes of untreated VF, a mechanical ventilator was connected to the animals; CCs on animals were performed to a depth of 5cm (IIa, n = 6) or a depth of 3cm (IIb, n = 6) with a 5-minute duration. Hemodynamic parameters were collected at the initiation and at beginning of the second and fifth minutes after VF.

In Group III (n = 20), VF was induced 30-minutes after animal preparation. After 3 minutes of untreated VF followed by 2 minutes of CCs (depth of 5cm) period with mechanical ventilation, animals were defibrillated at 100J biphasic. Then the animals which had ROSC (n = 18) were randomly assigned to three different CPR strategies: CCs were started again at the seventh minute and stopped at the twelfth minute in Group IIIa (n = 6) (to a depth of 5cm) and Group IIIb (n = 6) (to a depth of 3cm); in Group IIIc (n = 6), there was no other treatment. Hemodynamic parameters were collected at initiation and at the second, fourth, sixth, ninth and thirteenth minutes after VF. The standard of ROSC we used were: the return of a measurable pulse and blood pressure, an abrupt sustained increase in P_ET_CO_2_ (typically ≥ 40mmHg), and spontaneous arterial pressure waves with intra-arterial monitoring [8].

In all groups, mechanical ventilation was maintained, except during untreated VF periods. All animals were provided with CCs at a target rate of 105 CCs/min. They were transfused normal saline at a speed of 10 ml/ (kg∙h) during the experimental protocol, and intravenously received pumped propofol at a speed of 2 mg/ (kg∙h). 10 minutes after parameters collection, all the animals were euthanized with potassium chloride.

### Outcome measurements

The following hemodynamic parameters were monitored: heart rate (HR), systolic arterial pressure (SAP), diastolic arterial pressure (DAP), mean arterial pressure (MAP) and coronary perfusion pressure (CPP). CPP was calculated by subtracting the mid-diastolic right atrial pressure from the mid-diastolic aortic pressure[9].

### Statistical analysis

Statistical analysis was completed using SPSS (Version 20.0). Normality of the continuous variables was assessed using the Skewness-Kurtosis test. Normally distributed continuous variables were described as mean±SD and compared by Student's T-test (Group I and Group II) or analysis of variance (Group III). Continuous variables that were not normally distributed were described as median (25%, 75%) and evaluated by the Kruskal-Wallis test. A two-tailed (two-sided) probability value of less than 0.05 was considered to be statistically significant.

## Results

**Fig 2:**
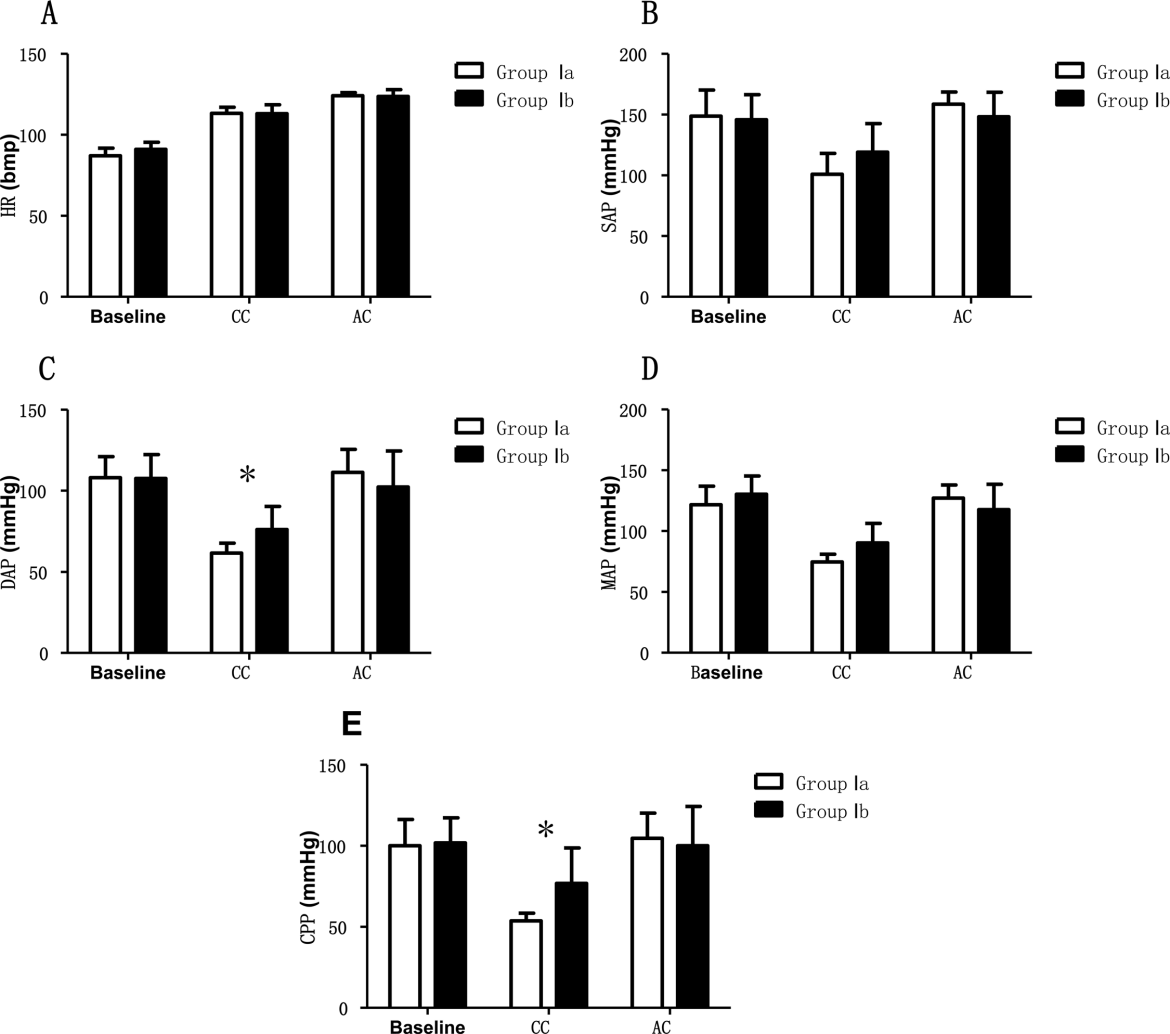
Hemodynamic values in non-arrested animals with chest compressions. CC = chest compressions; AC = after compressions. Group Ia = non-arrested with chest compressions (to a depth of 5cm); Group Ib = non-arrested with chest compressions (to a depth of 3cm); HR = heart rate; SAP = systolic arterial pressure; DAP = diastolic arterial pressure; MAP = mean arterial pressure; CPP = coronary perfusion pressure.*: statistically significant difference between treatment groups.

In Group I, the hemodynamic variables were the same at the pre-compression baseline in both Group Ia and Ib (p > 0.05). However, during and after CCs, the hemodynamic parameters changed (Table 1). There was statistical decrease in SAP, DAP, MAP and CPP when CCs were performed in both sub-groups (p < 0.05) (Fig 2). The DAP and CPP decreased more in Group Ia than in Group Ib at the same period (p < 0.05) (Fig 2).

**Table 1:** Hemodynamic parameters in non-arrested animals with chest compressions.

	Group Ia (n = 6)			Group Ib (n = 6)		
Parameters	Baseline	CC	AC	Baseline	CC	AC
HR (bpm)	87.0±4.9	113.3±3.8*	124.2±1.9†	91.0±4.4	113.0±5.6*	123.7±4.2†
SAP (mmHg)	148.8±21.4	100.8±17.3*	158.5±10.1†	145.7±20.7	119.2±23.4*	148.3±20.0†
DAP (mmHg)	108.0±13.0	61.7±6.1*	111.3±14.2†	107.5±14.8	76.0±14.3*‡	102.3±22.2†
MAP (mmHg)	121.6±15.2	74.7±6.4*	127.0±10.8†	130.2±15.0	90.4±15.8*	117.7±20.8†
CPP (mmHg)	100.1±16.2	53.7±4.6*	104.6±15.6†	101.8±15.6	76.8±21.9*‡	100.0±24.2†

*: statistically significant difference between sedation stage and chest compressions

†: statistically significant difference between chest compressions and after compressions

‡: statistically significant difference between treatment groups.

Group Ia = non-arrested with chest compressions (to a depth of 5cm); Group Ib = non-arrested with chest compressions (to a depth of 3cm); HR = heart rate; SAP = systolic arterial pressure; DAP = diastolic arterial pressure; MAP = mean arterial pressure; CPP = coronary perfusion pressure; CC = chest compressions; AC = after compressions.

**Fig 3:**
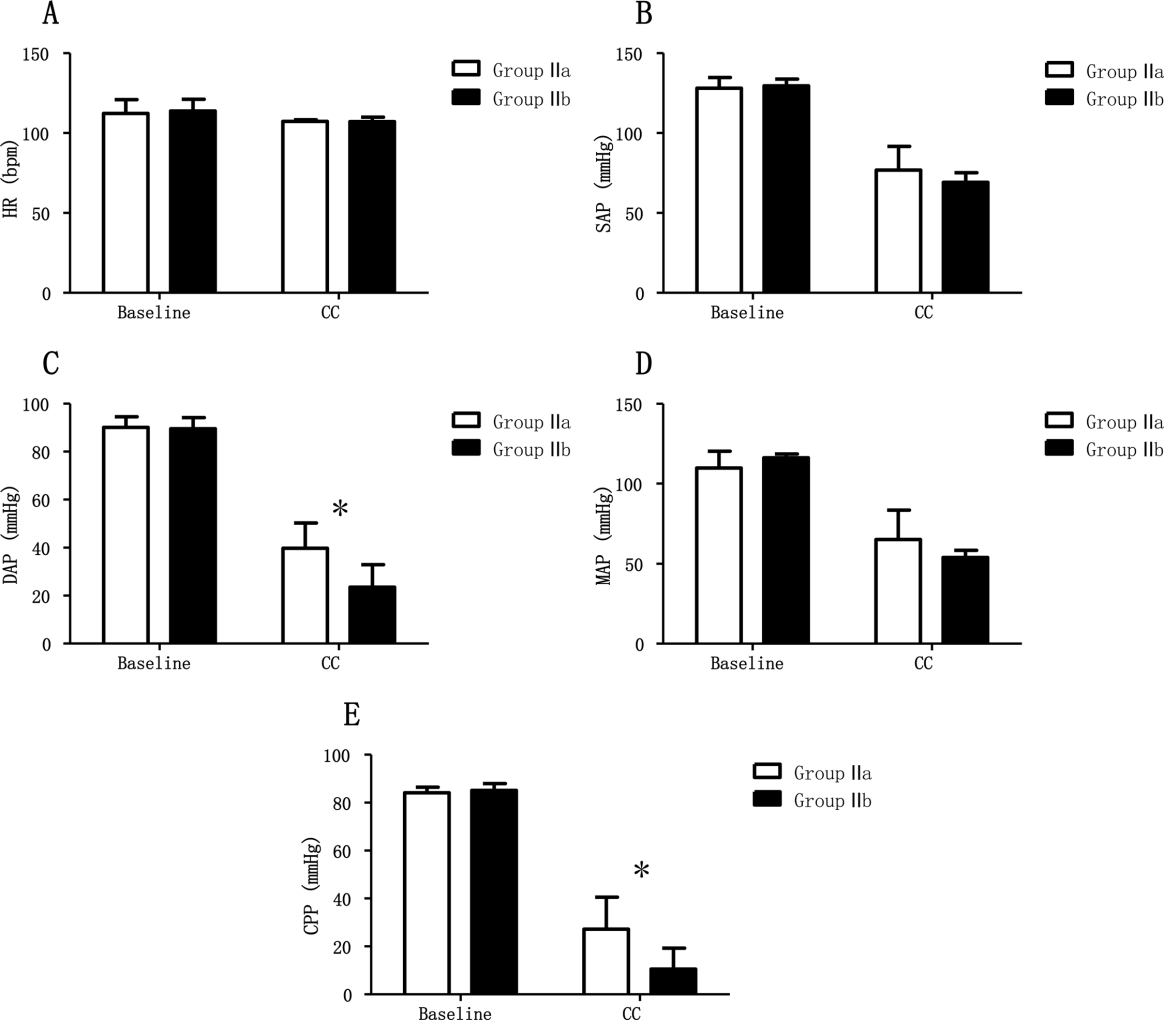
Hemodynamic values in arrested animals with chest compressions only. Group IIa = arrested with chest compressions only (to a depth of 5cm); Group IIb = arrested with chest compressions only (to a depth of 3cm); HR = heart rate; SAP = systolic arterial pressure; DAP = diastolic arterial pressure; MAP = mean arterial pressure; CPP = coronary perfusion pressure; CC = chest compressions. *: statistically significant difference between treatment groups.

In Group II, the hemodynamic variables were the same at the pre-compression baseline in both Groups Ia and Ib (p > 0.05). When CCs were performed, the hemodynamic parameters diverged (Table 2). The DAP and CPP were higher in Group IIa than in Group IIb during CCs (p < 0.05) (Fig 3).

**Table 2:** Hemodynamic parameters in arrested animals with chest compressions only.

	Group IIa (n = 6)		Group IIb (n = 6)	
Parameters	Baseline	CC	Baseline	CC
HR (bpm)	112.3±8.6	107.3±1.0	113.8±7.4	107.2±2.8
SAP (mmHg)	128.0±6.8	76.8±14.8	129.5±4.4	69.2±6.1
DAP (mmHg)	90.2±4.4	39.8±10.4	89.5±4.7	23.5±9.5*
MAP (mmHg)	109.8±10.5	65.2±18.2	116.2±2.4	53.9±4.5
CPP (mmHg)	84.2±2.3	27.2±13.4	85.2±2.8	10.5±8.8*

*: statistically significant difference between treatment groups.

Group IIa = arrested with chest compressions only (to a depth of 5cm); Group IIb = arrested with chest compressions only (to a depth of 3cm); HR = heart rate; SAP = systolic arterial pressure; DAP = diastolic arterial pressure; MAP = mean arterial pressure; CPP = coronary perfusion pressure; CC = chest compression.

**Fig 4:**
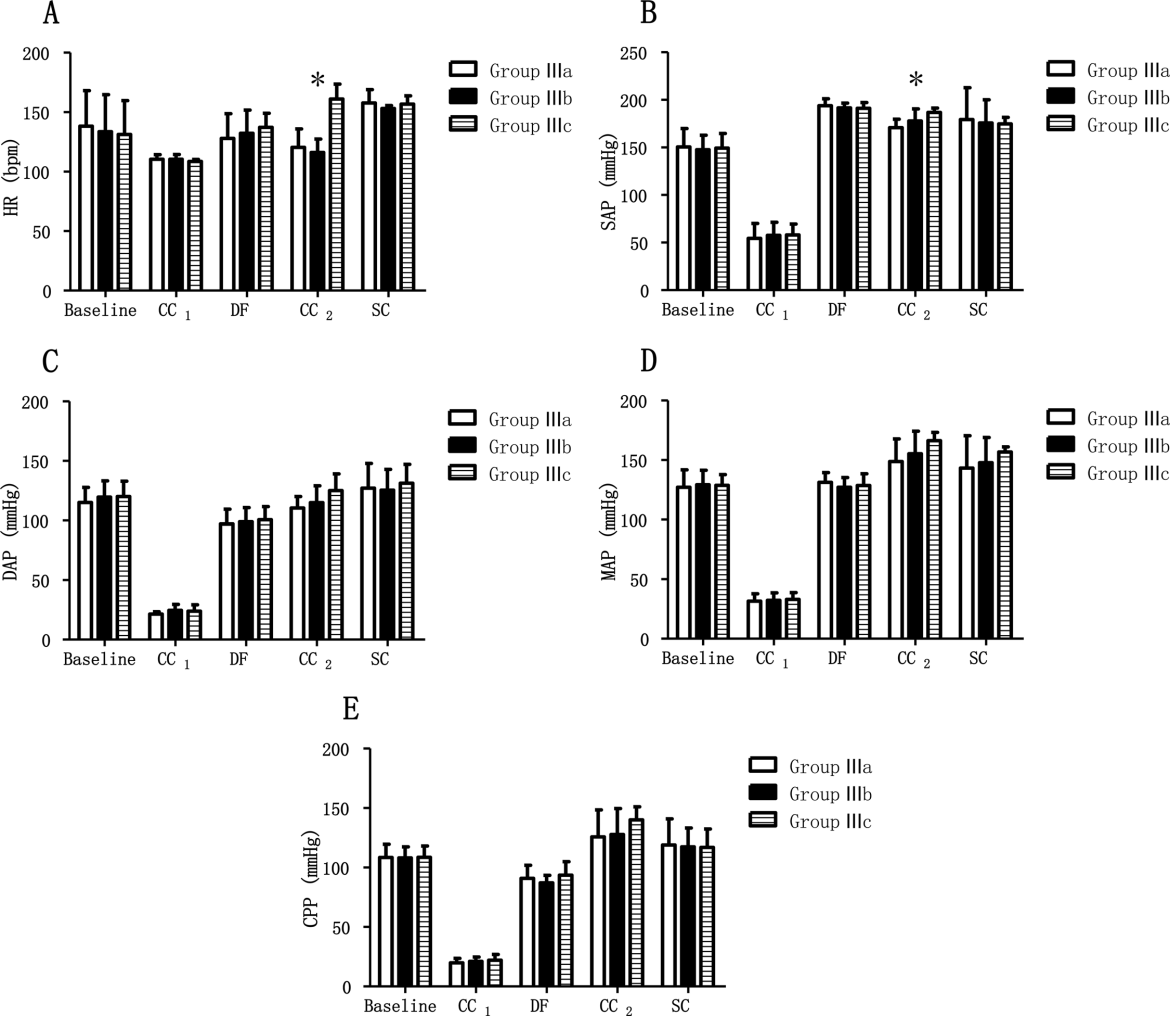
Hemodynamic values in arrested animals with chest compressions after ROSC. Group IIIa = compressions to a depth of 5cm continued after ROSC; Group IIIb = compressions to a depth of 3cm continued after ROSC; Group IIIc = chest compressions stopped after ROSC; CC1 = chest compressions to a depth of 5cm; DF = ROSC after defibrillation; CC2 = chest compressions in Group IIIa (5cm) and IIIb (3cm) and the same time in Group IIIc; SC = stop compressions in Group IIIa and IIIb and the same time in Group IIIc; ROSC = return of spontaneous circulation; HR = heart rate; SAP = systolic arterial pressure; DAP = diastolic arterial pressure; MAP = mean arterial pressure; CPP = coronary perfusion pressure. *: statistically significant difference between treatment groups.

In Group III, the hemodynamic parameters were similar at baseline, and when getting ROSC in all sub-groups (p > 0.05). However, the hemodynamic parameters differed when it came to the different treatments (Table 3). HR was lower in Group IIIa and IIIb than in Group IIIc during their respective periods of treatment (p < 0.05), and no hemodynamic differences were detected among the three groups after the treatment (p > 0.05) (Fig 4A). SAP was lower in Group IIIa than in Group IIIc during the period of different treatments (p < 0.05) (Fig 4B). Fig 4 shows hemodynamic parameter changes during the different treatments. There was no difference in any hemodynamic parameters among the sub-groups when CCs were finally stopped (p > 0.05).

**Table 3:** Hemodynamic parameters in arrested animals with chest compressions after ROSC.

Parameters	Group	Baseline	CC_1_	DF	CC_2_	SC
HR (bpm)	Group IIIa	138.2±29.9	110.3±3.9	127.8±20.8	120.3±15.6||	157.8±11.0‡,§
	Group IIIb	133.5±31.3	110.3±4.0	132.3±19.4*	116.0±11.4||	153.0±2.5‡
	Group IIIc	131.3±28.5	108.7±1.5	137.2±11.9*	161.0±12.6†	156.7±7.1§
SAP (mmHg)	Group IIIa	150.5±19.4	54.5±15.7	193.8±7.3*	170.7±9.0†,||	179.3±33.4
	Group IIIb	147.7±15.1	57.7±13.8	191.5±5.1*	177.8±12.8	175.7±24.4
	Group IIIc	149.2±15.5	58.0±11.4	191.7±6.0*	186.7±4.7†	174.7±7.0‡,§
DAP (mmHg)	Group IIIa	115.2±12.6	21.3±2.1	97.2±12.2*	110.3±9.8	127.0±20.9
	Group IIIb	119.5±13.8	24.7±4.8	98.8±12.0*	114.8±14.3	125.3±17.6
	Group IIIc	120.0±12.9	23.8±5.5	100.7±11.0*	125.0±14.0†	131.2±16.0‡,§
MAP (mmHg)	Group IIIa	127.0±14.8	31.5±6.2	131.7±8.2*	148.7±19.0	143.2±27.2
	Group IIIb	129.3±12.1	32.2±6.4	127.2±8.0*	155.2±19.1†	147.8±21.1
	Group IIIc	128.8±8.9	33.0±5.7	128.5±9.9*	166.2±7.1†,‡	156.7±4.4§
CPP (mmHg)	Group IIIa	108.3±11.3	22.0±4.9	90.8±11.1*	125.8±22.6†	118.8±22.1
	Group IIIb	108.2±9.2	19.8±3.8	87.0±6.4*	127.7±21.9†	117.3±15.9§
	Group IIIc	108.7±9.4	21.0±3.7	93.7±11.2*	140.0±11.1†	116.8±15.7‡

*: statistically significant difference between CC_1_ and DF

†: statistically significant difference between DF and CC_2_

‡: statistically significant difference between CC_2_ and SC

§: statistically significant different between stage DF and SC

|| statistically significant difference between treatment groups.

CC_1_ = chest compressions to a depth of 5cm; DF = ROSC after defibrillation; CC_2_ = chest compressions in Group IIIa (5cm) and IIIb (3cm) and the same time in Group IIIc; SC = stop compressions in Group IIIb and IIIc and the same time in Group IIIa; ROSC = return of spontaneous circulation.

Group IIIa = compressions to a depth of 5cm continued after defibrillation; Group IIIb = compressions continued to a depth of 3cm after defibrillation; Group IIIc = chest compressions stopped after defibrillation; HR = heart rate; SAP = systolic arterial pressure; DAP = diastolic arterial pressure; MAP = mean arterial pressure; CPP = coronary perfusion pressure.

## Discussion

CPR is a life-saving intervention and the cornerstone of resuscitation from cardiac arrest [2, 8]. As the latest CPR guidelines recommend, the key element of CPR is high quality CCs, emphasized by the phrase “push hard and push fast” and resuming CCs immediately for 2 minutes after defibrillation to minimize interruptions [3]. However, once the patients have just achieved ROSC, continuous CCs may not benefit the patient’s hemodynamics.

Our study showed that in animals with spontaneous circulation (Group I), CPP and arterial pressure decreased dramatically with CCs, and increased when CCs were stopped. The deeper the depth of CCs was, the greater the CPP decreased.

To do CCs or not is the question when someone collapses without cardiac arrest. Studies have shown that it is safe for bystanders to initiate CCs on out-of-hospital cardiac arrest patients, which might increase the survival rate of cardiac arrest patients [10, 11]. Some researchers reported that patients who suffered sudden collapse without cardiac arrest did not sustain any obvious damage and sustained no visceral organ injury occurred after bystander CPR [12]. One study showed that almost half of cardiac arrests were not detected in out-of-hospital cardiac arrest patients [13].

High quality CCs remain the key part of CPR for cardiac arrest patients. Studies have shown that cardiac output achieved by high quality CCs might only reach 1/4 to 1/3 of the normal circulation [3]. Successful adult resuscitation is more likely when CPP is > 20 mm Hg and when DAP is >25~30 mm Hg [14–16]. In this study, CPP and DAP reached such a goal in Group IIa but not in Group IIb. This result reflected the importance of high quality CCs during CPR.

VF is the most common reason for cardiac arrests, which is best treated with immediate defibrillation [3]. In this study, most animals had ROSC after defibrillation. At the early stage of post-ROSC, physiological parameters such as arterial pressure and CPP decreased when CCs were performed compared with those that did not receive CCs. It seemed that CCs might disturb the hemodynamics in the early post-ROSC condition. After defibrillation, the animals got ROSC in Group III. HR increased gradually in Group IIIc, but was interrupted by CCs in Groups IIIa and IIIb. The increasing HR was probably due to physiologic compensation after ROSC, the electric stimulation, and the pain response after CCs. While in Groups IIIa and IIIb, HR was interrupted by CCs, according to the frequency of CCs.

In order to minimize the interruption of CCs, the 2010 AHA/ERC CPR guidelines recommended providing 5 cycles (approximately 2 minutes) of CPR immediately after electric defibrillation without any pulse or rhythm check. However, if ROSC happens within 2 minutes, the artificial circulation provided by CCs would interrupt the spontaneous circulation. In this study, we found that these two kinds of circulations were not synergistic. The cardiac pump mechanism relies on a sequence of physiologic processes. On one hand, the rates and rhythms of spontaneous circulation and artificial circulation would most likely not be the same, and, on the other hand, even if the rates and rhythms did coincide, artificial compressions couldn’t physically match the systolic and diastolic periods of spontaneous circulation. In the cardiac cycle, the atria’s and ventricle’s systole and diastole are underway sequentially [17], and there is rarely a pure systolic or diastolic phase. With CCs’ simultaneous effect on the whole heart, the heart’s systolic and diastolic function is negatively affected. CCs are therefore not helpful for spontaneous circulation in general, let alone after ROSC, even leading to re-fibrillation [6, 7]. Osorio, et al. observed ventricular capture when CCs were performed [18], which created long–short cycles of activation leading to life threatening arrhythmias [19]. Berdowski’s study showed a relationship between the onset of CCs and VF recurrence [7]. We wonder if it is best to perform 2 minutes of CCs after defibrillation without checking for ROSC?

CCs have the highest priority when it comes to victims suffering sudden collapse, no matter the existence of cardiac arrest or not. However, a CPR team in the hospital that has sufficient equipment to better monitor a patient’s circulation could choose a different approach instead of starting CCs immediately, in order to achieve more precise treatment for every patient. In our view, after the first 2 minutes of CCs after defibrillation, if the victims had ROSC without stable circulation, high quality CCs should be performed continuously; if the victims had a return of stable spontaneous circulation, continuous CCs should be stopped. Here “stable” substitutes for a continuous circulation state, which maintains oxygen delivery to vital organs. While an "unstable" circulation does not last for >2 minutes, and thus needs continuous compressions. This is more relevant clinically when, for instance, pulseless electrical activity (PEA) alternates repetitively with spontaneous circulation. Once the victims had ROSC, the electrical activity of the myocardium recovered earlier than mechanical contractions. Electrical activity acquired from an electrocardiogram wouldn’t reflect myocardial contractility and hemodynamics. Therefore, recognition of spontaneous circulation during CPR seems to be extremely important.

At present, methods to monitor the patient’s physiological response to resuscitative efforts mainly include invasive hemodynamic data (e.g. CPP) and P_ET_CO_2_ [20, 21]. Xue JK et al suggested that shockable rhythms, CPR duration ≤15 minutes and total adrenaline dose ≤5 mg were favorable predictors of ROSC [22]. During CPR, myocardial blood flow was primarily determined by CPP [9, 15, 23, 24]. CPP positively correlated with myocardial blood flow, ROSC, and 24h survival in multiple studies [25–28]. However, the monitoring of CPP is difficult to achieve rapidly in emergent cases. P_ET_CO_2_ correlated well with cardiac output during CPR, being a prognostic indicator for survival [29, 30]; however, it could be transiently altered by giving IV sodium bicarbonate [31]. Therefore, we believe approaches that use non-invasive physiologic data are the future in peri-arrest situations, where early detection of ROSC might improve hemodynamics.

### Study limitations

The sample size of our study was small, so there was no statistical difference between groups in some parameters. Second, our study was performed on animal models. The phenomenon observed was an indirect reflection of the real changes which occur in human beings. Third, in Group I the stable baseline may not imitate the situation in all collapses. However, we believe it represented those patients with a stable circulation post-collapse who did not need CCs. There might be other reasons responsible for a victim’s sudden collapse, including bradyarrhythmia and hypotension. Further study should be made to make it clearer how CPR impacted hemodynamics in these situations. Fourth, without an invasive direct evaluation of coronary flow, CPP was indirectly estimated. Finally, during CCs the thermodilution PiCCO method was not available. We tried to measure pulse contour cardiac output (PCCO) by analysis of the pulse contour curve, finding the value inaccurate. Therefore, the parameter of PCCO wasn’t included.

## Conclusions

Chest compressions might be detrimental to hemodynamics in the early post-ROSC period. Chest compressions had a positive effect on circulation in cardiac arrest, but a negative impact on circulation for those returning to spontaneous circulation after resuscitation. The deeper the CCs were, the greater the positive effect during cardiac arrest was and the greater the negative effect after ROSC. These findings emphasize the importance of detecting the point of ROSC.

## Supporting Information

S1 Dataset This file includes all the data in the Manuscript. (XLSX)
